# Role of Vitamin D Supplementation in Heart Failure Patients With Vitamin D Deficiency and Its Effects on Clinical Outcomes: A Literature Review

**DOI:** 10.7759/cureus.10840

**Published:** 2020-10-07

**Authors:** Vishal Busa, Ahmed Dardeir, Suganya Marudhai, Mauli Patel, Sharathshiva Valaiyaduppu Subas, Mohammad R Ghani, Ivan Cancarevic

**Affiliations:** 1 Internal Medicine, California Institute of Behavioral Neurosciences & Psychology, Fairfield, USA; 2 Internal Medicine/Family Medicine, California Institute of Behavioral Neurosciences & Psychology, Fairfield, USA; 3 Internal Medicine, Richmond University Medical Center, New York, USA; 4 Neurology, California Institute of Behavioral Neurosciences & Psychology, Fairfield, USA

**Keywords:** vitamin d deficiency, heart failure, chf, congestive heart failure, vitamin d supplementation, vitamin d, long-term clinical outcomes, cholecalciferol, 25 hydroxyvitamin d, 25(oh)d

## Abstract

Vitamin D deficiency has become a global pandemic affecting approximately one billion people worldwide. Much attention has been paid to the association of low serum 25-hydroxyvitamin D (25(OH)D) levels and various chronic diseases, especially heart failure (HF). A clear role of vitamin D deficiency has been established, with increased mortality and morbidity in heart failures. However, previous randomized control trials have failed to show improvement in clinical outcomes with calciferol supplementation in these patients. Therefore, it is still unclear whether calciferol therapy can be added to the standard care in congestive heart failure (CHF) patients with deficiency. Hence, to evaluate the role of vitamin D supplementation in CHF patients with low serum 25(OH)D, we conducted an extensive search in the PubMed and Google Scholar databases using various combinations of keywords. All potentially eligible studies that evaluated the effects of vitamin D supplementation on clinical outcomes in HF patients were retrieved and extensively studied. We also checked the references of all eligible studies to identify additional relevant publications. In this study, we reviewed various mechanisms of vitamin D affecting the cardiovascular system and examined the impact of deficiency on heart failures in terms of mortality and hospitalizations. In conclusion, vitamin D supplementation has failed to improve the clinical outcomes in HF patients. The possible long-term benefits of supplementation cannot be excluded. Therefore, for future clinical trials, we recommend considering large sample sizes, longer follow-up durations, along with optimal dosage and appropriate dosing frequency.

## Introduction and background

Over the last two decades, vitamin D deficiency is a major topic of debate even though a remarkable amount of research has been done in the past. Indeed, according to recent research, 90% of physicians believe that they overprescribe calciferol supplements, considering the lack of side effects [1]. This overprescribing is due to the lack of specific guidelines for screening and treating patients with low serum cholecalciferol levels. Nearly one billion people worldwide are affected by either a vitamin D deficiency (<20 ng/ml) or insufficiency (21-29 ng/ml) [2]. A 2018 study that took data from the National Health and Nutrition Examination Survey (NHANES) estimated that the prevalence of vitamin D deficiency was 28·9% [3]. Researchers are aware that the role of vitamin D is not just limited to bone health, however, its role outside bone health is poorly understood. Research has shown that vitamin D deficiency has a negative impact on morbidity and mortality outcomes in patients who have chronic illnesses such as heart disease, diabetes mellitus, cancer, pulmonary hypertension, and auto-immune disorders [4-5]. Unfortunately, none of the current evidence was able to explain the association between vitamin D deficiency and these chronic diseases. A study by the Agency for Health Research and Quality reviewed nearly 250 studies and concluded that the results of these studies are inconsistent, making it difficult to establish a possible link between vitamin D and health outcomes [6]. Although a few studies have found a statistically significant reduction in all-cause mortality with supplementation of 25-hydroxyvitamin D (25(OH)D), further evaluation of these results did not show such reduction [7-8].

Among these chronic diseases, cardiovascular disease is the most common cause of death in the world and accounts for 30% of deaths, leading to a huge burden on health care systems [9]. Although there are a variety of cardiovascular diseases affected by vitamin D deficiency, congestive heart failure is the major one, accounting for the healthcare burden in terms of hospitalizations, healthcare usage, morbidity, and mortality primarily affecting middle and old age people [10-11]. As of 2017, almost 6.5 million adults have heart failure and contribute to one in every eight deaths [12]. To be more specific, outcomes in these patients can be changed and, in some cases, can prevent life-threatening events with simple lifestyle changes such as the intake of foods rich in vitamin D, dietary supplements, and exposure to sunlight.

A recent study reported that vitamin D deficiency increases the risk of hospitalization and mortality in these patients, but it is still unclear whether supplementation improves the outcome or not [13]. Results from the current literature have shown a wide range of variations and inconsistencies [14-19]. Thus, the effectiveness of supplementation is still unclear. Therefore, no specific guidelines were made for the inclusion of vitamin D therapy in the standard care of HF patients.

From this review, we are trying to assess the benefits of giving vitamin D supplements to CHF patients and its effects on clinical outcomes of CHF patients with low vitamin D. This study aims to summarize the results from previous studies, compare the conclusions, and finally draw inferences on whether vitamin D supplements improve the outcomes. We are also trying to summarize the underlying pathogenesis and mechanism of cardiac dysfunction in these patients. This summary of evidence will provide an update for current clinical practice; it will also help to counsel HF patients regarding misconceptions of vitamin D use and provide recommendations for future researchers.

## Review

Effects of vitamin D on the cardiovascular system and its role in heart failure patients

**Figure 1:**
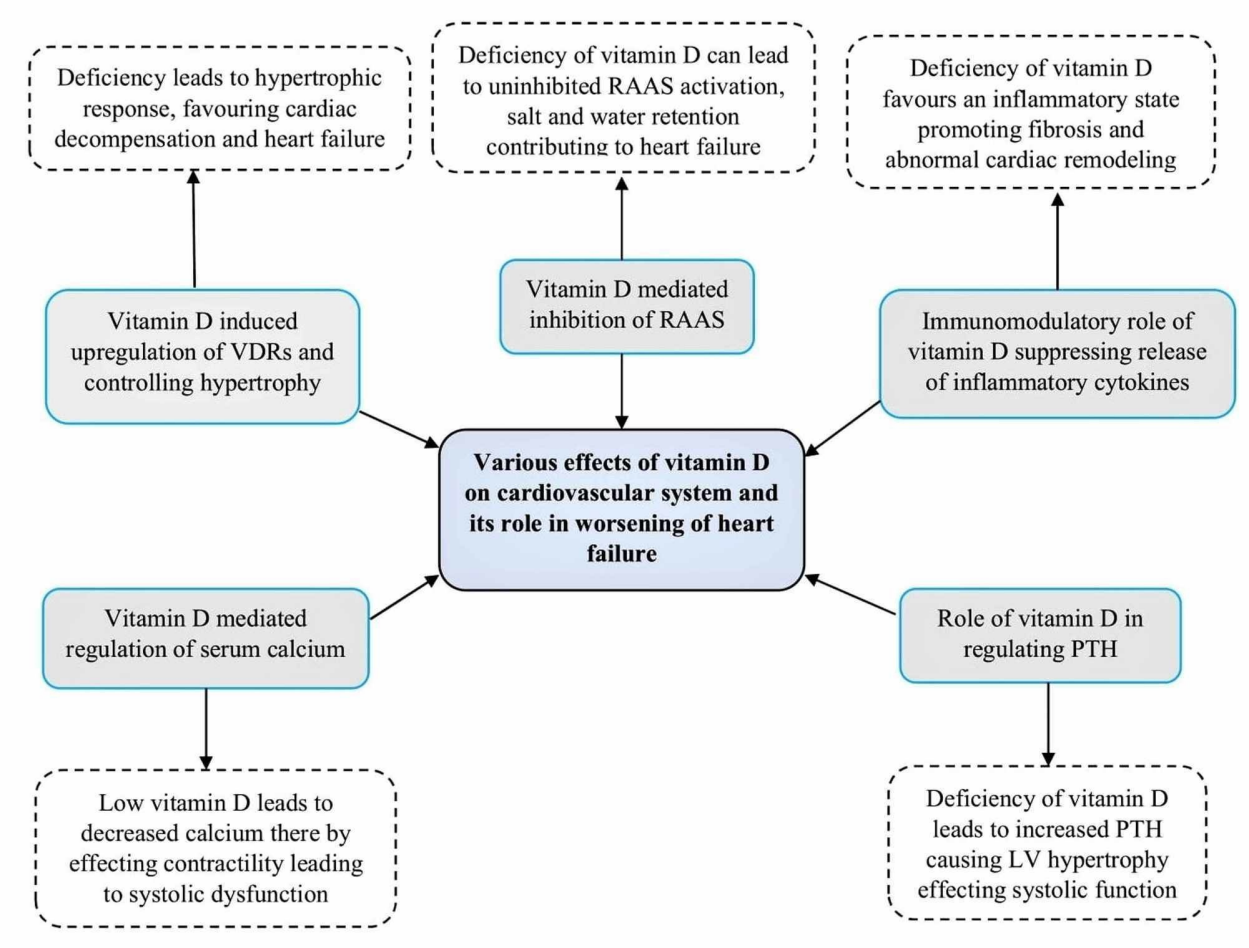
Various mechanisms involved in the pathophysiology of vitamin D deficiency-associated effects in heart failures RAAS=Renin Angiotensin Activation System, VDRs=Vitamin D Receptors, PTH=Parathyroid Hormone

Extensive research has established a clear role of 25-hydroxycholecalciferol in promoting the health of the cardiovascular system. Of the various functions of vitamin D, regulation of the Renin-Angiotensin activation system (RAAS) is the major cardiac protective action. Therefore, the deficiency can lead to uninhibited RAAS activation and contribute to the worsening of heart failure by the retention of salt and water. Data from a study conducted by Resnick et al. have shown an inverse relationship between vitamin D and serum renin levels. This relationship is mainly due to the suppression of the renin transcription with the help of vitamin D receptors (VDRs) [20]. Coming to the defensive role of cholecalciferol in controlling cardiac hypertrophy, in-vitro evidence has shown that VDRs in cardiac myocytes and fibroblasts are upregulated as a counter-regulatory mechanism to hypertrophy [21]. The absence of this role favors hypertrophic response leading to cardiac decompensation and heart failure. Another important role of vitamin D is its immunomodulatory mechanism, which decreases inflammatory effects by promoting anti-inflammatory T-helper cells like Th2 cells and inhibiting Th1 and Th17 cells that are responsible for inflammatory cytokines like interleukin (IL)-1, IL-6, and tumor necrosis factor-alpha (TNF-a). Few studies also reported that vitamin D suppresses the release of pro-inflammatory cytokines [22]. Furthermore, a negative correlation was found between serum 25(OH)D levels and these cytokines, resulting in an inflammatory state in deficient patients [23]. Vitamin D also regulates the metabolism of calcium and parathyroid hormone (PTH) and, in fact, these two also play a major role in cardiac contractility and remodeling, respectively. Low 25(OH)D levels lead to decreased calcium thereby effecting contractility leading to systolic dysfunction. In addition, deficiency may cause an increase in PTH, which, in turn, causes myocardial fibrosis and hypertrophy affecting LV systolic function. So, fluctuations in vitamin D can lead to alterations in serum levels of calcium and PTH, thus affecting the function of cardiac muscle indirectly. Taking into consideration all these different actions of 25-hydroxycholecalciferol on cardiovascular health, researchers believe that improving the vitamin D status in deficient patients may improve the clinical outcomes and overall mortality in heart failures. However, few recent clinical trials have found that there was no added advantage even with supplementation of vitamin D in these patients [14-17]. Therefore, the role of vitamin D as standard therapy in CHF patients is still unclear. But there are few studies that have shown some positive impacts on these patients appealing to researchers in need of further studies in the future to have a clear picture. Figure 1 below gives an overview of different mechanisms that are affecting cardiac muscle either directly or indirectly.

Role of vitamin D supplementation in CHF patients

Several investigators have attempted to examine the benefits of vitamin D supplementation in CHF patients and their results have suggested few recommendations for the current standard of care. A variety of regimens (such as high dose and low dose) for different durations (long-term and short-term) and different frequencies (daily, weekly, and monthly) were used in several clinical trials.

A recent study evaluated the effects of short-term vitamin D supplementation in heart failure patients for eight weeks [24]. They studied its relation to blood pressure (BP) and physical activity and concluded that supplementation has failed to improve BP and the Six-Minute Walk Test (6MWT) [24]. However, these results should be taken with caution, as the sample of this study was very small, and therapy was given for a short period without maintenance [24]. In contrast to this, a long-term clinical trial was conducted for three years on 400 HF patients who received daily 4000 IU with all-cause mortality as the primary endpoint [15]. No difference in mortality was observed between the treatment and control groups, with 19.6% of deaths in the treatment group and 17.9% of deaths in the placebo group, concluding its importance in advanced HF [15]. Their data also revealed no difference in hospitalizations, the need for cardiac resuscitation, and transplantation [15]. Furthermore, they noticed a greater need for mechanical circulatory support implants in the intervention group indicating caution with respect to long-term vitamin D supplementation [15]. In fact, this was the only RCT that provided evidence for the detrimental effects of moderately high-dose cholecalciferol in HF patients. They cited high plasma calcium concentrations as a reason for the adverse effects in these patients [25]. Coming to the limitations, this study has restricted their analysis to male patients only, and further, it could not prove the causal association [25]. A clinical trial was conducted by Scragg and his colleagues to examine whether a monthly high-dose vitamin D supplementation can prevent cardiovascular disease in the general population regardless of their serum 25(OH)D status [26]. A total of 5108 participants were selected, and half of them were randomly given oral D3 in an initial dose of 200,000 IU followed by a monthly 100,000 IU for a median of 3.3 years. Their primary outcome revealed no purpose of giving supplements, and it did not prevent any cardiovascular disease (CVD) events [26]. They stated that this non-significance may be due to their monthly regimen, however, a daily or weekly regimen can be more effective [26]. This suggests the importance of dosing frequency. To understand its effects better, Witte et al. conducted a randomized controlled trial with 229 patients with CHF and vitamin D deficiency [27]. Participants received either 4,000 IU of oral D3 or placebo for one year, the results showed clinically significant improvements in ejection fraction, LV dimensions, and volumes highlighting the role in the reversal of cardiac remodeling [27]. But supplementation had no effect on their primary endpoint of 6MWT [27]. Similar results were also found in another RCT conducted by Dalbeni et al., in addition to an increase in ejection fraction, the study also reported a beneficial effect on systolic blood pressure [28]. But when compared to Witte et al., the major drawback of this study was its small sample of 23 CHF patients and supplements given for only a short duration of six months [28].

Coming to the effects of vitamin D supplementation on serum renin levels, a study by Schroten et al. reported that there was a drastic decrease in renin levels in the treatment group [29]. They concluded that this reduction in renin cannot be translated as improved outcomes in these patients, as the absolute reduction was small [29]. But from the fact that sustained elevation in plasma renin activity (PRA) is an independent factor that predicts adverse outcomes in HF patients [30], we can consider reduced PRA as a positive interaction. They found no significant effect on natriuretic peptides that correlate with the severity of CHF [29]. The short duration of six weeks could be a limitation of this study. Another study showed a dramatic increase in serum 25(OH)D levels within six months of weekly high-dose therapy with a proportional decrease in PTH [31]. However, this increase could not be correlated clinically, as they found no difference when compared to the placebo [31]. The relatively small sample size of 30 subjects and short duration of six months were the drawbacks of this study. A unique study from 2011 tried to evaluate optimal treatment options for the adverse effects of vitamin D deficiency in heart failure patients [32]. In this study, weekly high-dose cholecalciferol of 50,000 IU along with daily calcium was supplemented. Their major finding was a decrease in plasma 8-isoprostane, which is a marker of oxidative stress and lipid peroxidation [32], and this reduced oxidative stress was clinically correlated with improved LVEF (Left Ventricular Ejection Fraction). Nevertheless, this study had its own limitations, first, the subjects recruited were restricted to African Americans with heart failure and vitamin D deficiency and followed up for 14 weeks only. Second, as the treatment regimen includes both cholecalciferol and calcium, their results cannot be applied to cholecalciferol alone, and, importantly, they did not have a control group to compare the findings [32].

A compelling amount of evidence has shown that inflammation plays a major role in the pathogenesis of HF. Few studies tried to evaluate the effects of supplementation on inflammatory markers [17,33]. A study published by Witham et al. found no significant difference in TNF-a levels in the treatment group when compared to placebo [17]. No changes were noticed in renin or aldosterone levels, but they found a clinically significant drop in brain natriuretic peptide (BNP) levels when compared to placebo, demonstrating the effects of vitamin D on the cardiovascular system [17]. Although BNP levels were reduced, they found no improvement in ejection fraction [17]. Another randomized control trial by Schleithoff et al. assessed the role of 25-hydroxycholecalciferol in reducing pro-inflammatory markers [33]. A higher level of IL-10 (anti-inflammatory) and lower levels of TNF-a (pro-inflammatory) were noticed, thereby improving the background inflammatory state in CHF patients [33]. Table 1 summarizes several clinical trials that evaluated the role of vitamin D supplementation in CHF patients.

**Table 1:** List of clinical trials that evaluated the role of vitamin D supplementation in CHF patients CHF=Congestive Heart Failure, 6MWT=6 Minute Walk Test, MCS=Mechanical Circulatory Support, LVEF=Left Ventricular Ejection Fraction, HR=Hazard Ratio, PTH: Parathyroid Hormone, TNF-a=Tumour Necrosis Factor-alpha, IL=Interleukin, BNP=Brain Natriuretic Peptide, ANP=Atrial Natriuretic Peptide, f/b=Followed By, 25-OHD=25-Hydroxyvitamin D, IU=International Units

Study details (year)	Follow-up	Vit D dose	Results
1. Hosseinzadeh et al. 2020 [24]	8 weeks	50,000 IU weekly	No improvements were found in BP and 6MWT when compared to the placebo. Levels of vitamin D were increased significantly in the intervention group. With short-term no improvement in HF patients’ physical activity consistent with previous studies.
2. Zittermann et al. 2017 [15]	3 years	4000 IU daily	No significant effect was found with supplements in advanced HF patients. The study also concluded that group on vitamin D had a greater need for MCS implants in the intervention group with HR 1.96 (1.04–3.66). No difference was found between the intervention and the placebo group in terms of mortality with HR 1.09 (0.69-1.71). Data also suggests caution with prolonged supplementation of high doses.
3. Scragg et al. 2017 [26]	3.3 years	200 000 IU f/b 100 000 IU monthly	Monthly high dose vitamin D did not prevent cardiovascular disease with HR 1.02 (0.87-1.21). Results also suggest that there was no purpose of supplementation in relation to heart failure with HR of 1.19 (0.84-1.68). This study also stated that high dose Vit D can be less effective than weekly or daily supplements in preventing a cardiovascular event.
4. Turrini et al. 2017 [16]	6 months	300,000 IU f/b 50,000 monthly	Treatment of deficiency did not influence the final outcome when compared to the placebo group. Supplements improved functional capacity and PTH levels at 3 months but this was not observed at 6 months. Concludes baseline vitamin D levels did not affect the functional capacity.
5. Witte et al. 2016 [27]	1 year	4000 IU daily	Results showed a significant improvement of cardiac function showing a mean change of +6.7 % LVEF (3.20-8.95) on echocardiogram. 1-year supplementation of daily Vit D did not improve 6-minute walk distance. It also has beneficial effects on LV structure and function in patients on current standard medical therapy.
6. Dalbeni et al. 2014 [28]	6 months	4000 IU daily	Results showed that there was a significant improvement in EF of 6.71% in elderly patients with HF and vitamin D deficiency. Therapy also improved SBP after 6 months from 129.6 to 122.7 mm Hg, but no significant variations were found on other parameters.
7. Schroten et al. 2013 [29]	6 weeks	2000 IU daily	Short term supplementation improved the Vit D levels from 48 nmol/L to 80 nmol/L. Results showed Plasma renin activity decrease from 6.5 ng/mL per hour (3.8-11.2) to 5.2 ng/mL per hour (2.9-9.5) in 6 weeks. No significant changes were seen in natriuretic peptides (BNP, ANP) and other markers of fibrosis.
8. Boxer et al. 2013 [30]	6 months	50,000 IU Weekly	High dose Vit D improved serum 25-OH D levels from baseline 19.1 ± 9.3 ng/ml to 61.7 ± 20.3 ng/ml. Vitamin D supplements did not improve physical performance for patients with HF despite a good increase in serum 25-OHD
9. Zia et al. 2011 [31]	14 weeks	50,000 IU weekly for 8 weeks f/b 1400 IU daily	Although a small patient population, results suggest improvement in secondary hyperparathyroidism, oxidative stress, and ventricular function (LVEF). Serum PTH was reduced at 14 weeks from 104.8 to 73.8 pg/mL, Plasma 8-isoprostane a marker of oxidative stress was reduced at 14 weeks to 117.8 from 136.1 pg/ml. With baseline EF of 24.3 ± 1.7% at entry was improved to 31.3 ± 4.3%.
10. Witham et al. 2010 [17]	20 weeks	100 000 IU and in the 10^th ^week	Supplementation did not improve functional capacity or quality of life in heart failure patients with vitamin D insufficiency. B-type natriuretic peptide levels decreased in the treatment group when compared with placebo
11. Schleithoff et al. 2006 [32]	9 months	1000 IU daily	Significant treatment effects were observed, parathyroid hormone levels were significantly decreased when compared to the baseline. Anti-inflammatory cytokine IL-10 was significantly higher in the intervention group at 9 months and pro-inflammatory cytokine TNF-a was increased in controls but remain constant in the therapy group. Vitamin D reduced the inflammatory state in CHF patients and might be a new anti-inflammatory agent in the future. But during follow-up at 15 months, the survival rate did not differ significantly.

Role of Vitamin-D as a predictor for risk of hospitalization and poor clinical outcomes

A considerable amount of literature has established the association of vitamin D deficiency and poor clinical outcomes in patients with HF. The majority of these studied the outcomes in terms of hospitalizations, risk of mortality, impact on LVEF, and effect on physical activity. A recent study reported vitamin D deficiency as an independent risk factor for hospitalization in patients with CHF [13]. Furthermore, this risk was more consistent in frail veterans when compared to non-frail veterans. An important aspect of this study was that its results failed to show any relationship between mortality and deficiency [13]. The main reasons for this lack of effect on mortality may be due to the small sample size and short follow-up period [13]. Another interesting study tried to determine the role of 25(OH)D levels on the clinical outcomes of HF patients undergoing cardiac resynchronization therapy (CRT) [34]. They concluded that serum calciferol levels less than 24.12 ng/ml has a significant impact on heart failure patients [34]. Deficient patients were more likely to show a lack of response to CRT and deficiency also predicts long-term mortality in these patients. This evidence convinced that the monitoring of vitamin D levels in HF patients could be of greater clinical significance by identifying high-risk groups. Similarly, another clinical trial also tried to demonstrate a response to CRT in HF patients with vitamin D deficiency [35]. Although the sample size was small, their results indicate that adequate levels of cholecalciferol can significantly improve the functional capacity of HF patients after undergoing CRT [35]. To understand its effects better, a key study from 2019 studied the pattern of mortality rates and the risk of hospitalizations in HF patients with respect to vitamin D status [36]. Findings from this study demonstrated a significant increase in rates of cardiovascular hospitalizations, but no association was found in terms of mortality, ejection fraction, and diastolic dysfunction [36]. Another longitudinal study examined the reason for increased hospitalizations and mortality rates in vitamin D-deficient HF patients [37]. Data from this study revealed no association with the risk of hospitalizations, however, they found significantly higher mortality in deficient patients when compared to non-deficient subjects [37]. This study also stated that a 2.72-fold increase in serum 25(OH)D levels can lower mortality by 14% [37]. A more interesting study attempted to assess the risk of readmission and infection rates after left ventricular assist device (LVAD) in HF patients with vitamin D deficiency [38]. They concluded that low vitamin D levels were independently associated with a higher risk of readmission and driveline infection risk [38]. Similarly, a cohort study investigated the relationship between serum vitamin D and the incidence of hospitalization in heart failure patients [39]. A greater risk of hospitalization was observed in deficient patients with a hazard ratio of 1.61 when compared to those with normal levels [39].

Due to the high prevalence of vitamin D deficiency in elderly patients, Porto et al. conducted a unique randomized clinical trial that evaluated the risk of heart failure in the elderly population [40]. A significant association was found between low levels of 25(OH)D and incidence of heart failure, this was significant especially in male patients and obese subjects. The higher incidence of heart failure in male subjects was strongly supported by the fact that vitamin D is positively associated with testosterone levels [41]. Testosterone, in turn, also has a protective effect on the myocardium, decreasing the risk of heart failure [42]. Thus, the combined effect of decreased testosterone levels and increased prevalence of vitamin D deficiency in elderly males led to a higher risk of HF [43]. In the same way, obese people are more prone to vitamin D deficiency, both obesity and vitamin D deficiency were independent risk factors for heart failure [44]. Another well-organized RCT from 2016 examined the role of vitamin D in predicting the rate of hospitalizations and mortality in HF patients [45]. This study finally concluded vitamin D as an independent predictor of hospitalizations and the risk of mortality [45]. The limitations of this study were the small sample size and small control group. Similar results were found by a study conducted by Liu et al. in 2012 and concluded that inadequate serum calciferol levels have a higher risk of mortality not only in HF patients but also in all cardiovascular diseases [46]. In another major study, vitamin D was found to be an independent factor in predicting survival rates in HF patients [19]. In this study, they evaluated the role of oral D3 supplementation and its effects on mortality. The treatment group was followed for 518 days and stated that vitamin D supplements improved the survival rates in these patients [19], thus highlighting the importance of including vitamin D therapy in the standard care of heart failures. Coming to the prognostic role of vitamin D in HF patients, a study by Liu et al. suggested that a low vitamin D concentration is associated with poor prognosis [47]. They also found high C-reactive protein (CRP) levels and increased renin activity in patients with low serum 25(OH)D [47]. Therefore, we can consider high levels of CRP and increased plasma renin activity as poor prognostic factors in HF patients. Table 2 summarizes studies that have evaluated the clinical outcomes in HF patients with vitamin D deficiency.

**Table 2:** List studies that evaluated the clinical outcomes in HF patients with vitamin D deficiency HF=Heart Failure, CRT=Cardiac Resynchronization Therapy, LVAD=Left Ventricular Assist Device

Study details	Measured outcomes	Risk estimates
1. Ugarriza et al. 2019 [13]	Hospitalization	1.8 (1.3-2.5)
	Mortality	0.83 (0.56-1.2)
	Hospitalization in frail subjects	1.7 (1.2-2.7)
	Mortality in frail subjects	0.84 (0.50-1.4)
2. Perge et al. 2019 [33]	5-year all-cause mortality	1.92 (1.02-1.45)
	Poor response to CRT	2.62 (1.01-6.25)
3. Nolte et al. 2019 [35]	Hospitalizations	1.74 (1.08-2.80)
	5-year mortality	1.55 (1.00-2.42)
4. Cubbon et al. 2019 [36]	Mortality	1.24 (1.05-1.46)
5. Obeid et al. 2018 [37]	Risk of readmission after LVAD	2.46 (1.07-5.77)
	Risk of driveline infection within 1 year after LVAD	6.18 (0.80-49.2)
6. Costanzo et al. 2018 [38]	Incidence of hospitalization	1.61 (1.06-2.43)
7. Porto et al. 2017 [39]	Incidence of heart failure	12.19 (4.23-35.2)
	Incidence of HF in males	15.32 (3.39-69.2)
	Incidence of HF in obese subjects	4.17 (1.36-12.81)
8. Belen et al. 2016 [44]	Hospitalizations (lower vs higher)	23.4% vs 7.3%
	Mortality (lower vs higher)	16.1% vs 1.2%
	Hospitalizations in higher levels	0.89 (0.84-0.95)
	Mortality in higher levels	0.83 (0.75-0.92)
9. Liu et al. 2012 [45]	Mortality	2.06 (1.01-4.25)
10. Gotsman et al. 2012 [19]	Mortality	1.52 (1.21-1.92)
	Mortality after supplementation	0.68 (0.54-0.85)

## Conclusions

In conclusion, our current review evaluated the role of vitamin D supplementation in CHF patients and explained various consequences of low serum 25-hydroxycholecalciferol in these patients. No significant difference was noticed with daily supplementation of vitamin D and similar results were observed with high-dose monthly or weekly supplements. We also found that deficiency of vitamin D is associated with increased risk of hospitalizations, mortality, and poor clinical outcomes. However, residual confounding lifestyle factors could also explain this inverse association. Possible long-term benefits from vitamin D supplementation cannot be excluded. To demonstrate effects on mortality and hospitalization more efficiently we require clinical trials with larger sample sizes along with longer follow-up durations. Therefore, further studies are essential in the future to comment on optimal dosing, frequency, and ideal serum target levels to improve outcomes in HF patients.
